# An Unusual Complication Encountered During the Extraction of the Femoral Nail: A Case Report

**DOI:** 10.7759/cureus.62413

**Published:** 2024-06-14

**Authors:** Nareshkumar S Dhaniwala, Kiran Saoji, Mukund N Dhaniwala, Ashutosh Lohiya

**Affiliations:** 1 Department of Orthopedics, Jawaharlal Nehru Medical College, Datta Meghe Institute of Higher Education and Research, Wardha, IND; 2 Orthopaedic Surgery, Dhaniwala Orthopedics and Dental Clinic, Nanded, IND

**Keywords:** precautions, complications, extraction, femoral nail, implant removal

## Abstract

Orthopedic implant removal is a common surgery performed either due to patient’s complaint of pain, dysfunction or infection or on doctor’s advice depending on the nature of the implant and its related future problems. The surgery may range from simple k-wire removal to difficult plate or intramedullary nail removal. Many unforeseen complications are experienced during implant removal, and occasionally, it results in failure of removal, fracture, prolonged per-operative bleeding and damage to nerves and vessels. We report here an unusual complication of coiling of a thick k-wire used during implant extraction surgery to prevent sinking of the nail and the difficulties in removing this coil and the nail, along with the precautions to be taken to avoid such difficulties.

## Introduction

Removal of implants is a common surgery in orthopedics because of the use of various intra- and extramedullary implants used for the fixation of fractures and for replacement surgery. Normally, the implants are removed long after the union of fracture or in cases of complications related to implants. Preferably, the implant should be removed by the surgeon who has applied it in the first surgery of fixation. Complications related to orthopedic implant surgery, may include infection, bleeding, damage to surrounding structures including nerves and blood vessels or persistent pain [1]. Implant removal in the upper and lower extremity normally causes notable improvement in dysfunction present earlier [2]. Greater bone contact with the implant increases stability and in such cases, removal may be more difficult, resulting in longer operative time and more intraoperative bleeding [3]. Implant removal may be difficult due to problems related to implant design and quality of the material used for its making, such as rounding or smoothening of the screw head or implant threaded end making it difficult to engage a screwdriver, extraction bolt or holding device. Many a times, removal of the implant can be far more difficult than insertion of the implant and its removal attempt may fail or cause additional complications. This case report describes an unusual complication faced during the extraction of the femoral nail and discusses the learning points of this experience.

## Case presentation

A 38-year-old male presented with the chief complaints of pain in the left thigh for three months and seropurulent discharge from a wound on the back of the thigh for four weeks. There was no history of fever, discharge of bony spicules, swelling or erythema in the area of the wound. The patient reported a grade 1 open fracture of the left femur that occurred 1.5 years back that was treated by intramedullary interlock nailing and cerclage wire at the district hospital. The postoperative period was uneventful and the patient had started full weight bearing three months after surgery.

**Figure 1:**
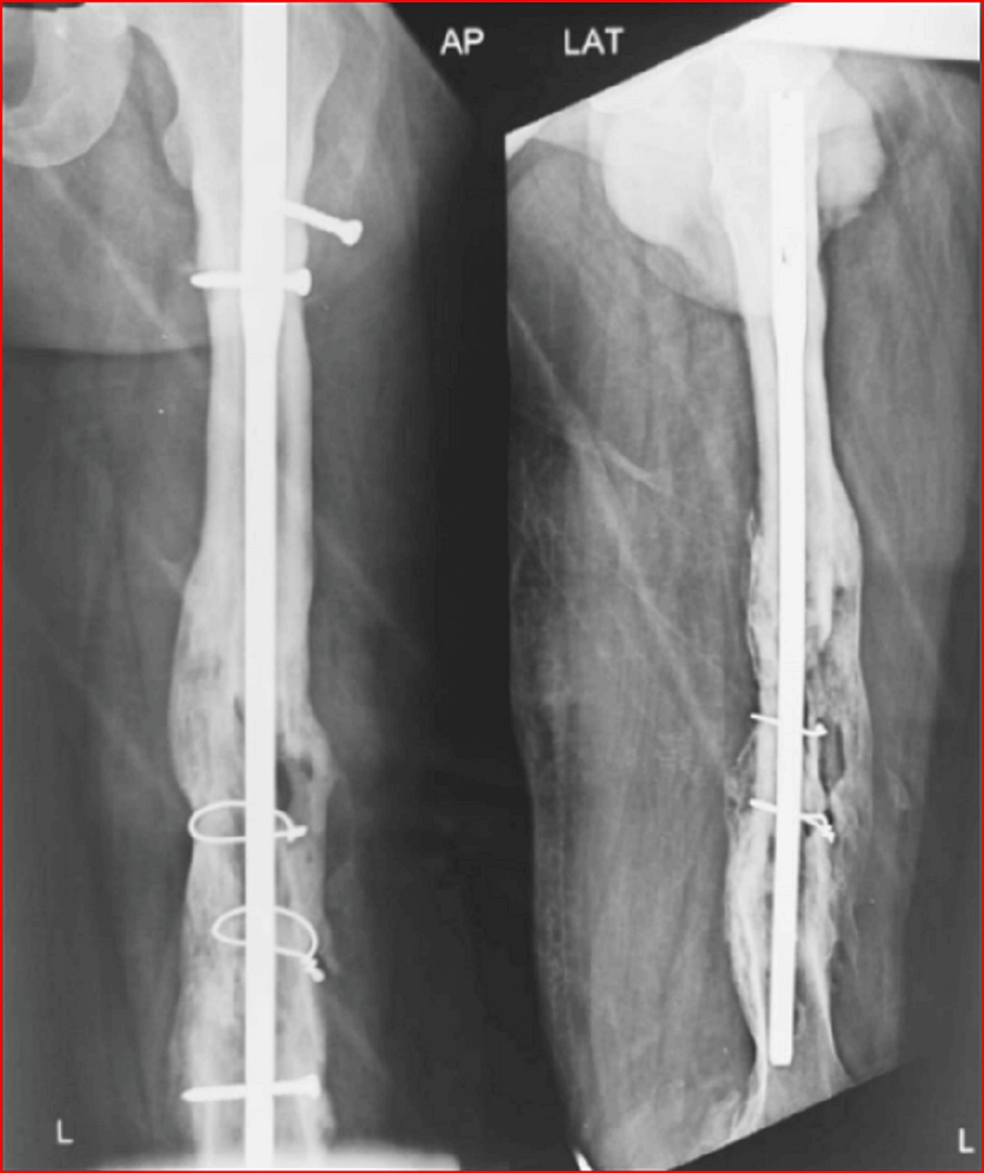
X-ray of the united femur fracture with an interlock nail, loose screws, cerclage wires and evidence of infection of the bone

A physical examination revealed a united left femur fracture with a normal hip and knee function and a sinus situated on the back of the mid-left-thigh. There was thickening of tissues near the sinus. An X-ray of the left thigh showed evidence of a segmental united fracture in the distal half of the femur with reasonable callus formation on medial and anterior aspects. There was an interlock nail in the bone with two interlocking bolts each proximally and distally. The proximal most screw was loose and had backed out partly (Figure 1). There were two cerclage wires with the tip embedded in the callus. There was lysis around the nail in the main fracture area. The proximal end of the nail and the distal bolts’ head were also buried in the bone.

Blood count of the patient showed white blood cells at 7800/cubic mm; ESR was 81 and CRP was 10. The culture of discharge from the sinus showed coagulase-positive *Staphylococcus* (methicillin-resistant *Staphylococcus aureus*, or MRSA). Sensitive antibiotics, in combination, were started and a decision to remove the implant was taken in view of the persistent pain, discharge from the sinus and the fracture totally united. With the patient in the right lateral position after spinal plus epidural anesthesia, the pyriform fossa of the femur was exposed and an entry was made with a bone awl under C-arm guidance. The proximal end of the nail was palpated and the universal extractor bolt was attached to it. It was confirmed by checking transmitted movements of the extractor to the thigh. The proximal interlocking bolts were removed easily. The distal interlocking bolts could be exposed after nibbling the overlying bone and were removed.

**Figure 2:**
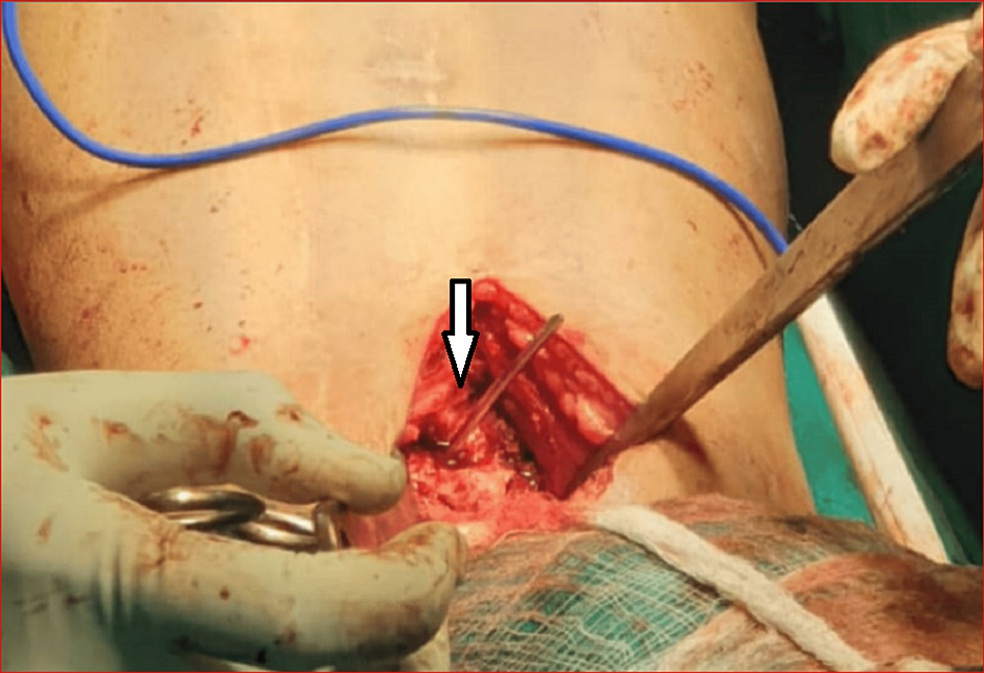
A blocking k-wire inserted in the distal interlock hole (white arrow)

On attempting to remove the nail by back hammering on the extractor, the universal bolt of the extractor got disengaged from the nail. As the proximal end of the femoral nail was buried in the bone, reengaging the extractor bolt to the nail end was failing repeatedly. It was also realized that the nail was loose in the medullary canal and could sink further and rotate. To prevent this unwanted sinking and rotation of the nail, a 3-mm thick Kirschner (k) wire was inserted in the distal interlocking hole of the nail (Figure 2).

**Figure 3:**
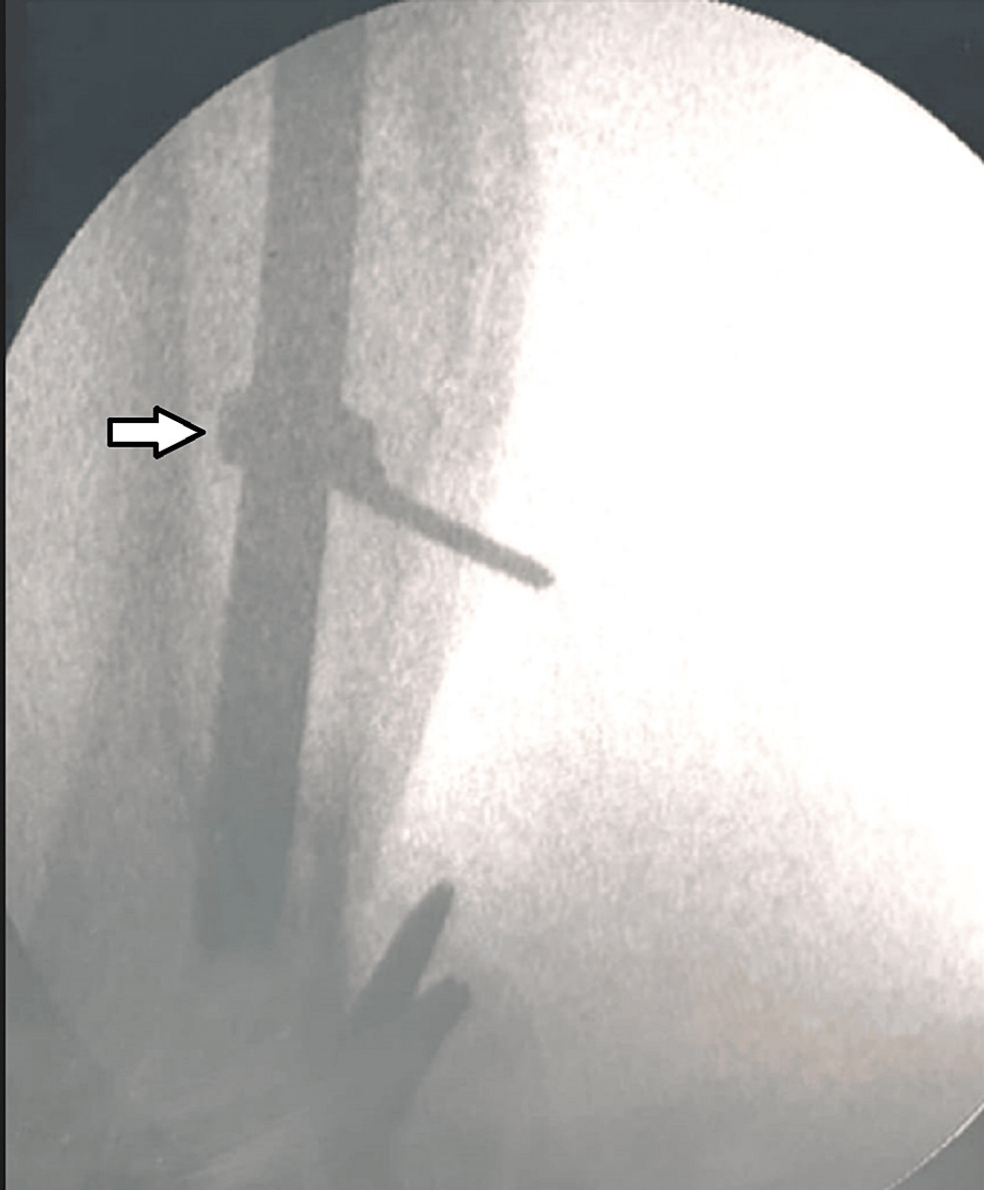
Fluoroscopy image of the k-wire partly coiled around the nail (white arrow)

The universal extractor bolt could finally be engaged to the nail end over a guide wire after many attempts and confirmed with the C-arm IITV (image intensifier TV) system. At this moment, it was noted that the blocking k-wire was reduced in length and was not coming out on attempts of pulling. On fluoroscopy, it was noticed that the k-wire in the distal interlocking hole had got coiled around the nail with two complete turns, during our attempts of reattachment of the extraction bolt (Figure 3).

**Figure 4:**
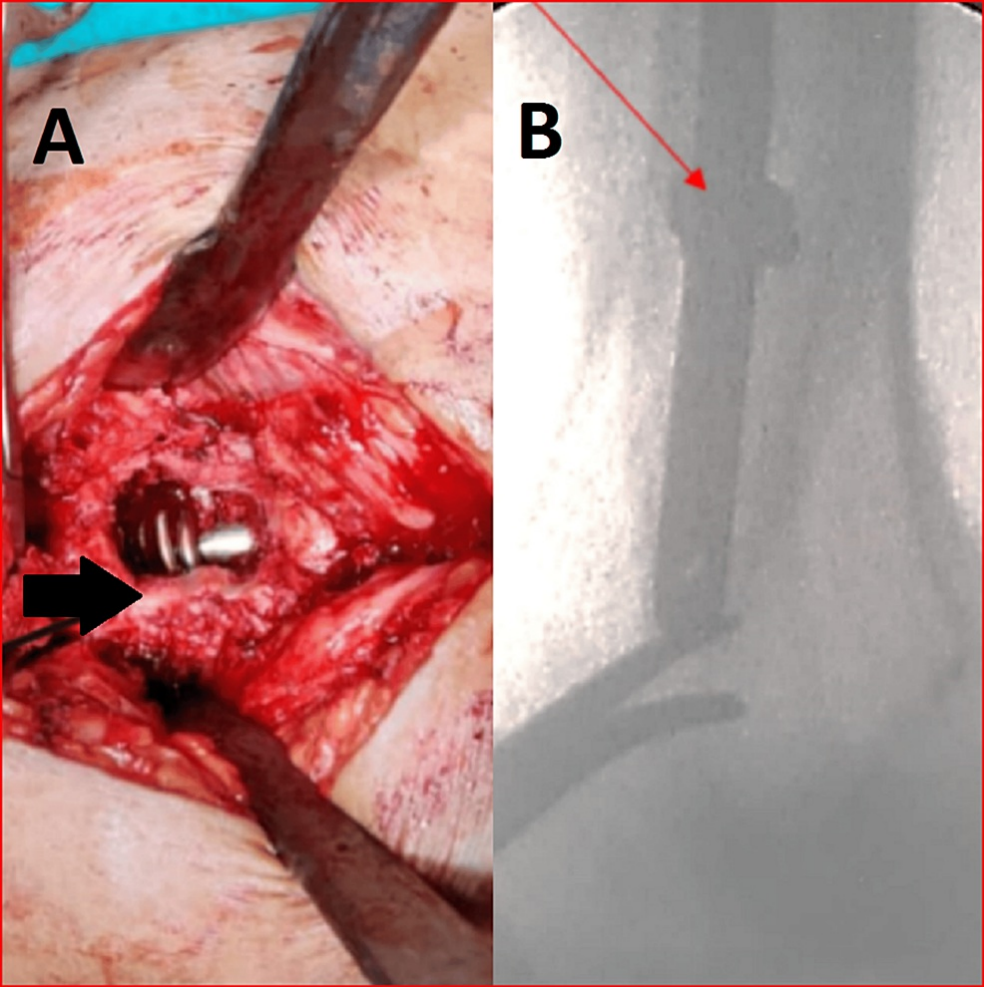
(A) Intraoperative view of the coiled k-wire around the nail and (B) its image on fluoroscopy

A window was made in the lateral cortex of the femur and the coils of the k-wire were visualized. Using a wire cutter, an attempt was made to cut the coils, but the blade of the wire cutter also broke. As the leading tip of the k-wire was traversing to the hole in the nail, it was impossible to extract the nail without removing the coiled k-wire. This k-wire now had two parts, one straight part outside the nail and the other coiled part around the nail. Finally, the straight portion of the k-wire could be cut as close to the nail as possible (Figures 4A, 4B).

**Figure 5:**
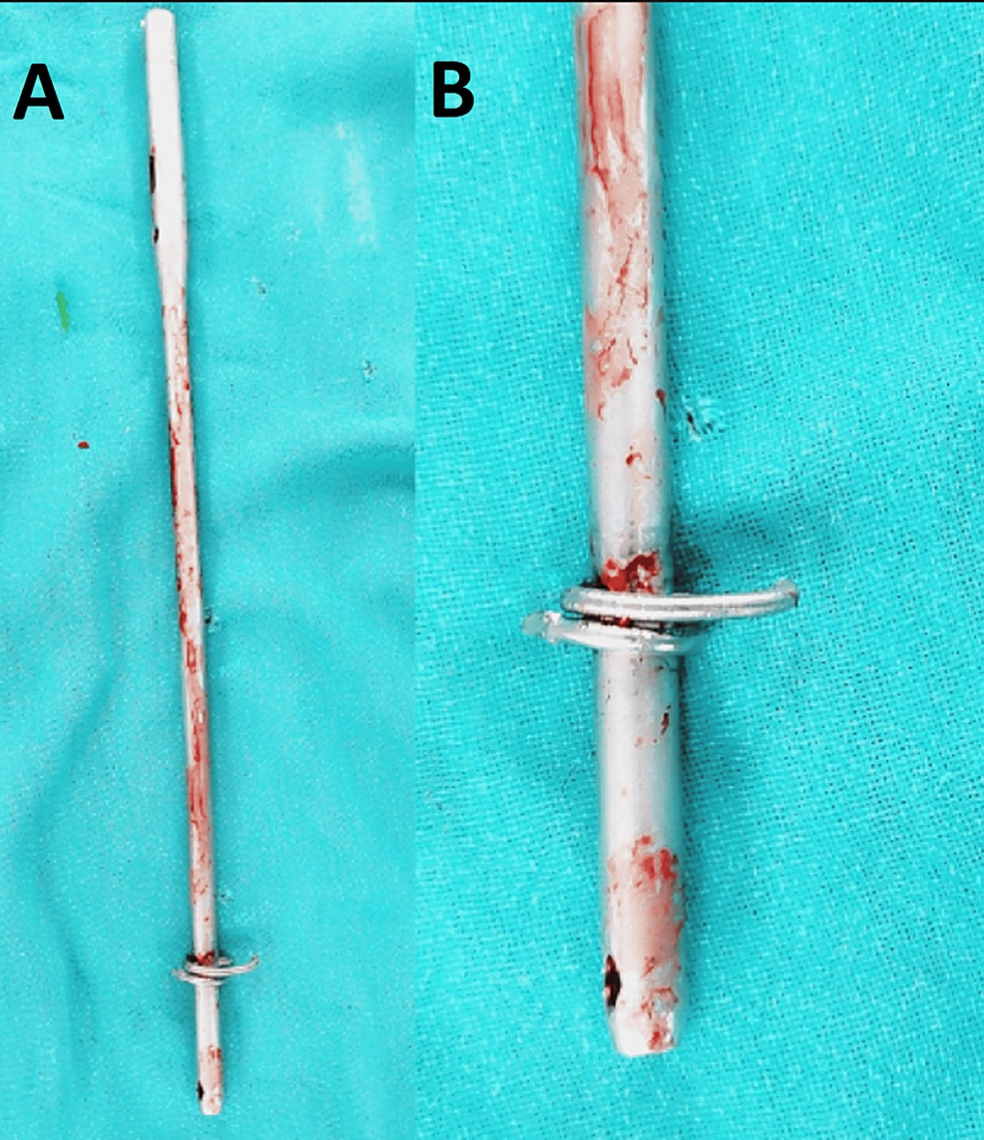
(A) Removed femoral nail with the coiled part of the k-wire inserted through the hole; (B) coiled k-wire

As there was a risk of the nail getting jammed in the medulla due to an increase in its size as a result of surrounding coils, removal of the nail was not attempted. Coil removal/separation was needed along with taking out of the intra-nail part of the wire. This could be achieved using the bone hook by which the intra-nail k-wire was brought out of the hole. Now the coiled part of the k-wire was held with the cockup forceps and the nail was removed by back hammering on the extractor rod. The coiled k-wire was removed through the lateral window in the femur (Figure 5).

The wounds were thoroughly irrigated and closure was done in the layers over a suction drain. The sinus tract was excised. The patient was kept non-weight bearing for four weeks and the sensitive antibiotics were given for six weeks postoperatively. The sinus healed completely and the pain disappeared, with the patient having a normal hip and knee function.

## Discussion

Various authors have studied implant removal surgery in different settings. The patient’s request and doctor’s recommendation are the two most frequent indications of implant removal. Symptoms of intractable pain, impaired function of the part and chronic infection in the area are other common indications for implant removal [1]. The complication rate reported in cases of implant removal is 10%, whereas improvement in function is seen in 72% cases [4]. A rapid, safe, minimally invasive and cost-effective method for percutaneous pedicle screw removal had been described by Ding et al. [5]. Gas gangrene following implant removal after the union of a tibial plateau fracture has been reported by Wang and Liu [6]. A retrospective study done at a single center from 2016 to 2022 noted hospitalization of more than a day in 44% cases and one-day hospitalization in 56% cases of implant removal besides a complication rate of 6% [7]. Intramedullary nail removal may become difficult due to implant design and formation of the bone at the tip of the nail and the amount of bone contact between the nail and the bone [8]. A greater bone contact with titanium increases implant stability making titanium nail removal more difficult than stainless steel nails in diaphyseal fractures of the tibia [3]. Implant removal done after a long gap may cause more difficulties in the surgery due to bone overgrowth, ingrowth and jamming of the implant as described by many authors [9]. A novel technique by drilling around the proximal end of the femoral nail without using fluoroscopy has been described by Misailidis et al. [10].

A descriptive cross-sectional study done at a tertiary care center found that implant removal surgery formed nearly one-fifth of the total orthopedic operations; implant removal was done as an elective procedure in 80% cases. More than 50% of patients undergoing implant removal were in the age group of 17 to 39 years [11].

Implant removal may be a simple and yet, at the same time, the most difficult surgery, more so when removal is done by a surgeon other than the surgeon for the index surgery or a less experienced surgeon. In the reported case, the coiling of the blocking wire happened during fixation of the extraction bolt because the nail was loose and the advancing tip of the k-wire was passing across the interlocking hole. The nail itself was acting as a lever for coiling the k-wire.

This case teaches us many points of practical importance that should be considered before any implant removal surgery. In cases of the proximal end of the nail buried in the bone, it is difficult to fix the extractor bolt. In such cases, an entry should be made with the bone awl and the proximal end of the nail be palpated with it. A guide wire should be inserted in the nail and the extractor bolt should be passed over the guide wire and tightened securely in the nail end. Secure and full threaded attachment of the extraction bolt to the nail’s threaded end is must to avoid disengagement of the extractor as happened in the reported case.

In the case of a need of blocking the interlocking hole, a thick Steinmann pin or a long interlocking bolt should be used instead of the k-wire, so that any possibility of coiling is prevented. The size and make of the implant should be checked prior to surgery and a specific implant extraction set should be made available. A senior surgeon should be available for help. Proper preoperative assessment of the patient and X-rays showing full details of the implant and bone are a must. A complete implant removal set along with the drill bit, nibbler, plier, wire cutter, bone hook, various screw drivers, osteotomes, bone cutter, bone awl and miscellaneous instruments should be available.

The patient must be explained the nature of the surgery and the difficulties expected. The possibility of failure of implant removal due to unforeseen circumstances must be explained and a written informed consent should be obtained. As a safe policy in the era of consumer protection act and litigation by a patient, it is preferred that the implant should be removed by the implant fixing surgeon as the surgeon knows all the details and difficulties encountered during the index surgical procedure. The operation notes should mention all details including the size, diameter and make of the implant along with the number of screws and other additional items used. A note regarding per-operative difficulties faced, if any, would help at the time of implant removal. The need of implant removal should be kept in mind at the time of implant fixation surgery.

Implant removal may require more time, may be difficult and might fail on many occasions due to the poor quality of the implant and manufacturing defects. The implants may undergo bending, breakage or fracture as well. Implants from standard companies using a certified material, following proper procedures in the manufacturing process and having permissions of the relevant quality control authorities regarding manufacture and standardization should only be used to avoid such problems.

## Conclusions

The reported case discusses the unforeseen problems encountered during the extraction of an interlocking femoral nail, namely, problems in fixing the extraction bolt, coiling of the k-wire (which was used for preventing nail sinking) around and into the nail and its management by innovative means of cutting the wire and removing its coiled part after the removal of the nail. It also points out the precautions and steps to be taken before going ahead with implant removal surgery.

Implant removal surgery should be considered more important and difficult than implant insertion surgery. A proper study of patient X-rays, implant design, status of screws and implant should be taken into consideration and a guarded prognosis should be explained to the patient. Availability of the instrument extraction set and guidance from a senior should be ensured in difficult cases.
